# Healthcare virtualization amid COVID-19 pandemic: an emerging new normal

**DOI:** 10.1080/10872981.2020.1780058

**Published:** 2020-06-12

**Authors:** Arun-Kumar Kaliya-Perumal, Usama Farghaly Omar, Jacquilyne Kharlukhi

**Affiliations:** a Lee Kong Chian School of Medicine, Nanyang Technological University Singapore, Singapore; b Department of Orthopaedic Surgery, Khoo Teck Puat Hospital, Singapore; c Department of Paediatrics, Sree Balaji Medical College and Hospital, Chennai, India

The COVID-19 pandemic has impacted us at various levels. Currently, we cannot predict how long this pandemic is going to last nor if there will be a second wave. Hence, it is necessary that we stay diligent on the safe distancing policy that is in practice in most institutions. In line with this policy, hospitals need to restrict patient numbers both in the outpatient clinics and inpatient wards. For this reason, hospitals are now encouraging virtual healthcare wherever possible, shedding the earlier hesitation to adopt the same [1,2].

Virtual healthcare is a common term for the various digital healthcare modalities that patients can use to seek medical advice. This not only includes the telehealth platforms (video, audio and instant messaging) that healthcare providers can use to remotely communicate with patients but also all other validated digital services for patient education and mentoring. Even though virtual healthcare was in practice before the pandemic, it was not in the limelight. Only radiologists and pathologists routinely reported from a distance, whereas others used telehealth platforms for follow-up and education of pre-diagnosed patients with a common chronic condition or for post-procedural monitoring that doesn’t require eliciting of signs. It was thought to be not suitable for first visits since a comprehensive physical examination cannot be performed.

Now that patient visits to hospitals must be restricted due to the pandemic situation, patients have started to adopt virtual healthcare technologies, especially telehealth platforms as a first line option to seek clinical care and healthcare providers are using this to perform a virtual triage using dialogue and questionnaires to shortlist who needs to be examined in person. Since most institutions are having a first-hand experience of how virtual healthcare works, we may move to a future with virtual healthcare as an integral part of health care infrastructure. However, since we do not know how this new normal is accountable medicolegally, healthcare providers need to act wisely in selecting patients for virtual healthcare.

Health care providers need to be familiar with the technology and receive orientation prior to adopting telehealth platforms; in addition, ‘health professionals have to comply with existing legislation, associated regulations, and the medical ethical guidelines adopted and followed in their country’ [3]. On the other hand, patients opting for virtual healthcare, especially for telehealth consultations need to be educated and reliable, as the information they provide is vital for making decisions. They should be aware that any misinformation or missed information could compromise their health and should understand the limitations of virtual healthcare and provide appropriate consent. Given the digital nature of virtual healthcare, there is also a threat to data confidentiality and security and this needs to be overcome.

Even though there are concerns regarding virtual healthcare, there are many advantages, including, but not limited to convenience, cost-effectiveness, timeliness of care and specialist access to people in remote areas [4]. These advantages outweigh the concerns. With more and more institutions adopting virtual healthcare, it is impending to become the new normal after this pandemic palliates [5]. This is something that this pandemic situation has made us effectuate after so many years of living with the required technology but being uncertain of its potential. It is time to overcome the challenges and be prepared for the virtual healthcare revolution.
